# COX-2 activation is associated with Akt phosphorylation and poor survival in ER-negative, HER2-positive breast cancer

**DOI:** 10.1186/1471-2407-10-626

**Published:** 2010-11-15

**Authors:** Sharon A Glynn, Robyn L Prueitt, Lisa A Ridnour, Brenda J Boersma, Tiffany M Dorsey, David A Wink, Julie E Goodman, Harris G Yfantis, Dong H Lee, Stefan Ambs

**Affiliations:** 1 Laboratory of Human Carcinogenesis, Center for Cancer Research (CCR), National Cancer Institute (NCI), NIH, Bethesda, Maryland (MD), USA; 2 Cancer Prevention Fellowship Program, Office of Preventive Oncology, NCI, NIH, Bethesda, MD, USA; 3 Radiation Biology Branch, CCR, NCI, NIH, Bethesda, MD, USA; 4 Gradient Corporation, Cambridge, Massachusetts, USA; 5 Pathology and Laboratory Medicine, Baltimore Veterans Affairs Medical Center, Baltimore, MD, USA

## Abstract

**Background:**

Inducible cyclooxgenase-2 (COX-2) is commonly overexpressed in breast tumors and is a target for cancer therapy. Here, we studied the association of COX-2 with breast cancer survival and how this association is influenced by tumor estrogen and HER2 receptor status and Akt pathway activation.

**Methods:**

Tumor COX-2, HER2 and estrogen receptor α (ER) expression and phosphorylation of Akt, BAD, and caspase-9 were analyzed immunohistochemically in 248 cases of breast cancer. Spearman's correlation and multivariable logistic regression analyses were used to examine the relationship between COX-2 and tumor characteristics. Kaplan-Meier survival and multivariable Cox proportional hazards regression analyses were used to examine the relationship between COX-2 and disease-specific survival.

**Results:**

COX-2 was significantly associated with breast cancer outcome in ER-negative [Hazard ratio (HR) = 2.72; 95% confidence interval (CI), 1.36-5.41; comparing high versus low COX-2] and HER2 overexpressing breast cancer (HR = 2.84; 95% CI, 1.07-7.52). However, the hazard of poor survival associated with increased COX-2 was highest among patients who were both ER-negative and HER2-positive (HR = 5.95; 95% CI, 1.01-34.9). Notably, COX-2 expression in the ER-negative and HER2-positive tumors correlated significantly with increased phosphorylation of Akt and of the two Akt targets, BAD at Ser136 and caspase-9 at Ser196.

**Conclusions:**

Up-regulation of COX-2 in ER-negative and HER2-positive breast tumors is associated with Akt pathway activation and is a marker of poor outcome. The findings suggest that COX-2-specific inhibitors and inhibitors of the Akt pathway may act synergistically as anticancer drugs in the ER-negative and HER2-positive breast cancer subtype.

## Background

Cyclooxygenase-2 (COX-2) catalyzes the conversion of arachidonic acid to prostaglandin E_2 _(PGE_2_) and enhances the metastatic phenotype of both breast cancer cells in vitro and breast tumors [1]. Increased COX-2 expression occurs early in breast cancer and can be detected in ductal carcinoma in situ [2], invasive breast carcinoma [3] and in metastatic lesions [4]. Recently, COX-2 expression has been associated with decreased disease-free survival in breast cancer [5], and breast cancer specific survival [6-8], suggesting that the inhibition of this enzyme has anticancer effects.

We have previously observed a significant association between COX-2 expression and Akt phosphorylation in breast tumors [9]. We also demonstrated the ability of PGE_2 _to induce phosphorylation of Akt in the ER-negative MDA-MB-231 breast cancer cells, and to a lesser degree in ER-positive MCF-7 breast cancer cells. The results indicated that COX-2 is a key modulator of Akt activation in breast cancer which is consistent with other published findings [10]. Additionally, it has been shown by others that administration of the COX-2 inhibitor, celecoxib, in murine mammary tumor models results in inhibition of Akt phosphorylation and enhanced induction of apoptosis [11].

In the current study, we hypothesized that COX-2 expression would be associated with poor breast cancer survival, and that the COX-2 effect on survival would be modified by the tumor ER and human epidermal growth factor receptor 2 (HER2) status and/or Akt pathway activation. A recent celecoxib anti-aromatase adjuvant trial did not find a clinical benefit for celecoxib, a COX-2-specific inhibitor, among ER-positive patients with advanced disease [12], while two other similar trials showed promising, albeit not significant effects of this drug when administered in combination with exemestane [13,14]. We investigated the association of COX-2 expression with disease outcome among ER-positive and ER-negative breast cancer patients. The results from our study suggest that COX-2-specific inhibitors could be more efficacious in ER-negative tumors than ER-positive tumors and may perhaps synergistically interact with Akt inhibitors in breast cancer survival.

## Methods

### Tissue collection

Paraffin-embedded (n = 248) tumor specimens were obtained from breast cancer patients that resided in the greater Baltimore area, as described [9]. Patients were recruited at the University of Maryland Medical Center (UMD), the Baltimore Veterans Affairs Medical Center, Union Memorial Hospital, Mercy Medical Center, and the Sinai Hospital in Baltimore between 1993 and 2003. All patients were identified through surgery lists and enrolled into the study prior to surgery. They signed a consent form and completed an interviewer-administered questionnaire. Clinical and pathological information was obtained from medical records and pathology reports. Disease staging was performed according to the tumor-node-metastasis (TNM) system of the American Joint Committee on Cancer/the Union Internationale Contre le Cancer (AJCC/UICC). The Nottingham system was used to determine the tumor grade. The collection of tumor specimens, survey data, and clinical and pathological information was reviewed and approved by the University of Maryland Institutional Review Board for the participating institutions (UMD protocol #0298229). IRB approval of this protocol was then obtained at all institutions (Veterans Affairs Medical Center, Union Memorial Hospital, Mercy Medical Center, and Sinai Hospital). The research was also reviewed and approved by the NIH Office of Human Subjects Research (OHSR #2248).

### Immunohistochemistry

IHC was performed as described previously [9]. Staining specificity was evaluated and shown with negative and positive control slides and, if available, with blocking peptides that were purchased from the manufacturer. Phospho-specific blocking peptides were available for phosphorylated Akt and phosphorylated caspase-9. To block phospho-Akt staining, blocking peptides from Cell Signaling for Akt Ser473 (#1140) and Akt Thr308 (#1145) were used. The specificity of these blocking peptides for phospho-Akt has been shown by the manufacturer. In brief, protein expression was evaluated using the following primary antibodies: 1:50 diluted monoclonal antibody (clone 33; no. 610204 (formerly C22420); BD Biosciences/Transduction Laboratories, San Diego, CA) for COX-2; 1:100 diluted rabbit polyclonal antibody (DakoCytomation) for HER2 (c-erbB-2); and ready-to-use monoclonal (Clone 6F11) antibody (Ventana Medical Systems, Tucson, AZ) for the estrogen receptor (ER); 1:25 diluted rabbit polyclonal antibody (no. 9277; Cell Signaling Technology, Beverly, MA) for phosphorylated Akt (Ser^473^); 1:80 diluted monoclonal antibody 244F9 (no. 4056; Cell Signaling Technology) for phosphorylated Akt (Thr^308^); 1:100 diluted rabbit polyclonal antibody (no. 9295; Cell Signaling Technology) for phosphorylated Bad (Ser^136^); 1:250 diluted rabbit polyclonal antibody (no. SC-11755; Santa Cruz Biotechnology, Santa Cruz, CA) for phosphorylated caspase-9 (Ser^196^), and the ready-to-use monoclonal antibody (Lab Vision Corp., Fremont, CA) for CD31. The IHC protocol to determine the tumor ER status followed guidelines for clinical laboratories to evaluate semi-quantitatively ER expression in formalin-fixed, paraffin-embedded tissue on a Ventana automated slide stainer for clinical assessment of a patient's ER status ("CONFIRM Estrogen Receptor" assay by Ventana). The IHC staining protocol for HER2 followed the DAKO HercepTest™ protocol. A combined score of intensity and distribution was used to categorize the immunohistochemical staining for protein expression with the exception of the ER IHC. Intensity received a score of 0 to 3 if the staining was negative, weak, moderate, or strong. The distribution received a score of 0 to 4 if the staining distribution was <10% positive cells, 10%-30%, >30%-50%, >50%-80%, and >80%. A sum score was then divided into four groups as follows: (1) negative = 0-1, (2) weak = 2-3, (3) moderate = 4-5, and (4) strong = 6-7. The ER status was scored negative/positive. The ER status was determined at the Department of Pathology, University of Maryland, according to the reference range set by the ChromaVision^® ^ACIS^® ^assisted quantitative image analysis software (Clarient Diagnostic Services, Irvine, CA), consistent with clinical guidelines. The HER2 status was determined using either the sum score system, as described above, or the score system according to the HercepTest™ protocol. However, additional FISH results to detect HER2 amplification were not available for this patient cohort. The quantification of the tumor MVD was performed on CD31-positive microvessels according to the method of Weidner et al [15]. Microvessels were counted per 200× field in the most vascular region of the tumor.

### *TP53 *mutational analysis

Tumors were screened for p53 mutations as previously described [16].

### Statistical analysis

Data analysis was performed using Stata/SE 10.1 (Stata Corp, College Station, TX) statistical software package. All statistical tests were two-sided, and an association was considered statistically significant with *P *values <.05. Spearman's rank correlation and logistic regression models were used for correlation analysis and to calculate odds ratios (ORs), respectively. Multivariable regression models were applied to calculate adjusted ORs. Survival was determined for the period from the date of hospital admission to the date of the last completed search for death entries in the Social Security Index (date of search: December 31^st^, 2006) for the 248 patients. The mean and median follow-up times for breast cancer survival were 71 months and 68 months, respectively (range: 12 to 166 months). A total of 89 (36%) of these 248 patients died during this period. We obtained information (National Death Index, death certificates) on the causes of death for the deceased patients and censored all patients whose causes of death, such as accidents, were not related to breast cancer (n = 11). The Kaplan-Meier survival method and the log-rank test of equality of survival function were used for univariate survival analysis. Cox regression was used for multivariable survival analysis to calculate adjusted hazard ratios. For the survival and logistic regression analyses, COX-2 expression was dichotomized into high and low. COX-2 IHC scores of moderate to strong were categorized as high and scores of negative to weak were categorized as low. The following covariates were included into the analyses: age at diagnosis (as a continuous variable), race/ethnicity (African-American versus European-American), TNM stage (categorized as ≤stage II versus > stage II), tumor grade (categorized as ≤grade 2 versus > grade 2), chemotherapy (yes/no), and p53 mutation (categorized as negative versus positive). In the univariate analysis, age at diagnosis, TNM stage, tumor grade, and p53 mutational analysis were significantly associated with disease outcome. Proportional hazards assumptions were verified by log-log plots and with the nonzero slope test of the scaled Schoenfeld residuals.

## Results

### Study population characteristics

**Figure 1:**
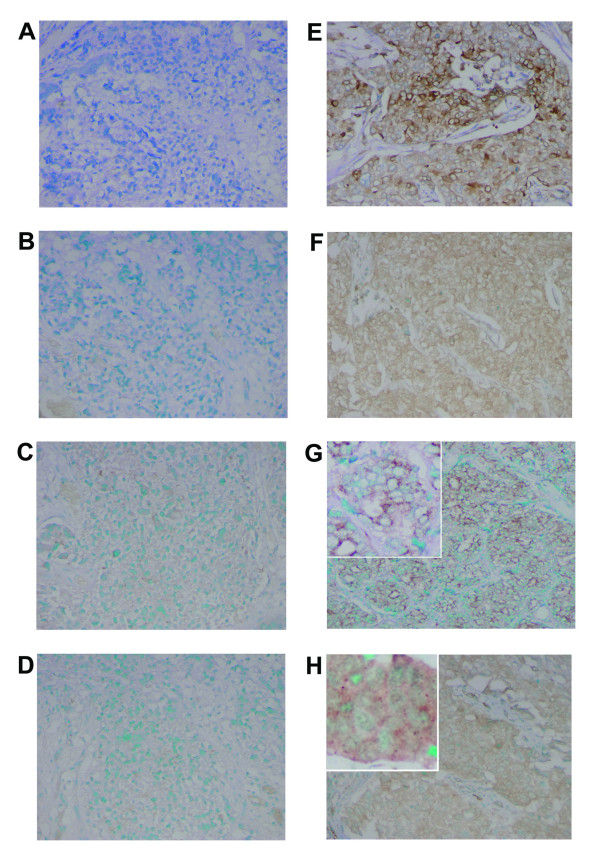
**COX-2 expression and phosphorylation of Akt at Ser473, BAD at Ser136 and caspase-9 at Ser196 in human breast tumors**. IHC analysis of two invasive carcinomas for COX-2 (A,E), Ser473-phosphorylated Akt (B,F), Ser136-phosphorylated BAD (C,G), and Ser196-phosphorylated caspase-9 (D,H). Tumor to the left was negative for all markers (A,B,C,D). Tumor to the right shows that COX-2 is expressed in the cytoplasm of tumor cells with some cells showing marked perinuclear localization of COX-2 protein (E). Immunostain intensity is moderate to strong. Same tumors shows increased phosphoryation of Akt in the cytoplasm and along the inner cell membrane (F), and moderate to strongly increased phosphorylation of BAD (G) and caspase-9 (H), with a distinct immunostain suggesting a partly mitochondrial localization of the phosphorylated proteins (insets). Magnification: 200×. Counterstain: Methyl Green.

**Figure 2:**
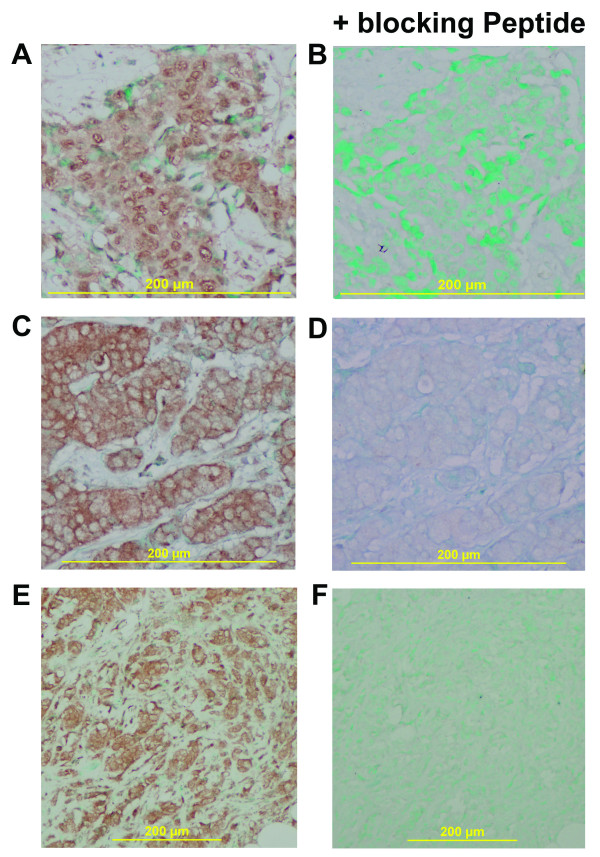
**Phosphorylation of Akt at Thr308 and Ser473 in human breast tumors**. IHC analysis of invasive ductal carcinomas for pAkt at Thr308 (A,B) and Ser473 (C-F). (A) Thr308-phosphorylated Akt is both cytosolic and nuclear in distribution. (B) The phospho-Akt (Thr308)-specific blocking peptide inhibits binding of the anti-phospho-Akt (Thr308) mouse monoclonal antibody. Panels C,E show the distribution of Ser473-phosphorylated Akt in tumor cells. (C) Phosphorylated Akt has a partly cytosolic distribution with a predominant staining at the inner cell membrane. Strongly increased Akt phosphorylation at Ser473 in another tumor (E). (D,F) Immunostaining for Akt Ser473 is blocked after pre-incubation with a phospho-Akt (Ser473)-specific blocking peptide. The IHC results are in agreement with the published literature that phosphorylated Akt can be detected in the cytoplasm, and that Ser473 phosphorylation occurs at the inner cell membrane and precedes Thr308 phosphorylation, which leads, as a second step, to membrane detachment and nuclear translocation of phosphorylated Akt [36-38]. Magnification: 100× for E,F; 200× for A,B,C,D. Counterstain: Methyl Green.

**Figure 3:**
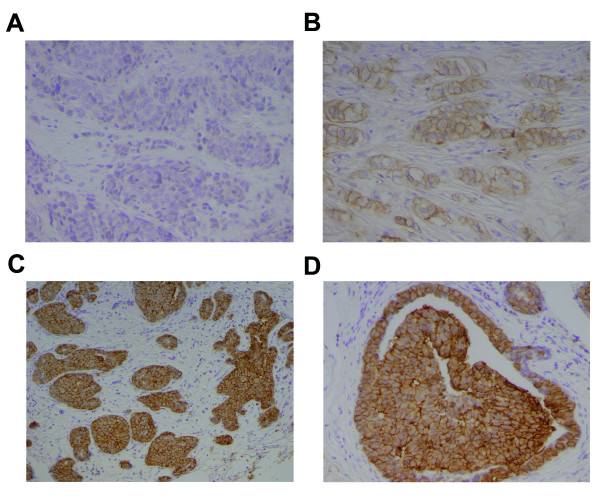
**HER2 overexpression in breast tumors**. Representative IHC staining for HER2 expression in invasive breast carcinomas (A-D). HER2 is predominantly membrane-bound (B-D). (A) Tumor that is negative for HER2. (B) Tumor with moderate HER2 staining (HercepTest™ 2+ staining). (C,D) Tumor with strong HER staining (HercepTest™ 3+ staining). Magnification: 100× for C; 200× for A,B,D. Counterstain: Hematoxylin.

We have previously reported a functional relationship between tumor COX-2 expression and Akt pathway activation in breast cancer [9]. To explore this finding further, we examined the relationship between tumor COX-2 expression and disease outcome in the same patient population from the greater Baltimore area. Patient characteristics and tumor marker expression are described in Table 1. Representative immunostains for COX-2, phosphorylated Akt, BAD, and caspase-9 are shown in Figures 1 &2 and for HER2 in Figure 3. TNM stage, tumor grade, tumor ER and HER2 status, patient's race/ethnicity, or age at diagnosis were not associated with COX-2 expression in the tumor. However, COX-2 expression was found to be inversely correlated with body mass index at hospital admission. After stratification by tumor ER status, this association was restricted to patients with ER-negative breast cancer (Spearman's correlation coefficient: -0.24; *p *= 0.024).

**Table 1:** Demographic and clinicopathological features of cases

		N	%
ER Status	Negative	102	41%
	Positive	145	59%
HER2 Status	Negative	106	43%
	Weak	48	19%
	Moderate	51	21%
	High	42	17%
TNM Stage	< = II	184	71%
	> = III	44	29%
Grade	1 or 2	108	50%
	3	107	50%
COX-2	Negative	75	30%
	Weak	83	34%
	Moderate	65	26%
	High	25	10%
pAkt Ser473	Negative	17	7%
	Weak	29	12%
	Moderate	59	24%
	High	142	57%
pAkt Thr308	Negative	14	6%
	Weak	24	10%
	Moderate	71	29%
	High	134	55%
pCaspase9 Ser196	Negative	38	15%
	Weak	30	12%
	Moderate	73	30%
	High	106	43%
pBAD Ser136	Negative	43	18%
	Weak	41	17%
	Moderate	76	31%
	High	85	34%
Survival	Alive	159	64%
	Death from breast cancer	78	32%
	Death from other causes	11	4%
Race	AA	143	58%
	EA	105	42%
p53 mutation	Negative	200	81%
	Positive	48	19%
Chemotherapy	No	99	43%
	Yes	132	57%

		mean ± SD	

Age at Diagnosis	(n = 248)	55.0 ± 13.9	
Body mass index^1^	(n = 236)	29.0 ± 8.1	
CD31	(n = 208)	49.1 ± 43.9	

^1 ^At time of hospital admission

### COX-2 expression predicts poor disease-specific survival in both ER-negative and HER2 expressing breast cancers

**Figure 4:**
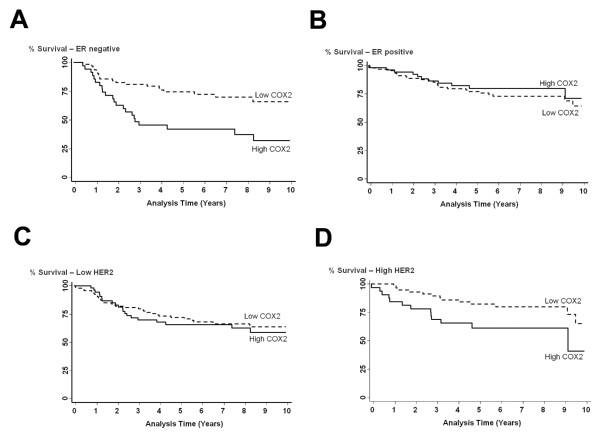
**Association between COX-2 and breast cancer survival by ER and HER2 status**. Kaplan-Meier cumulative breast cancer-specific survival curves of (A) ER-negative breast cancer patients by tumor COX-2 status (n = 98). The survival of patients with high COX-2 expression (n = 35) was significantly poorer than the survival of patients with low COX-2 expression (n = 63). Log-rank test: *p *= 0.001. (B) ER-positive breast cancer patients by tumor COX-2 status (n = 139). The survival of patients with high COX-2 expression (n = 51) was not significantly different from the survival of patients with low COX-2 expression (n = 88). Log-rank test: *p *= 0.483. (C) Low HER2 expressing breast cancer patients by tumor COX-2 status (n = 154). The survival of patients with high COX-2 expression (n = 54) was not significantly different from the survival of patients with low COX-2 expression (n = 100). Log-rank test: *p *= 0.601. (D) High HER2 expressing breast cancer patients by tumor COX-2 status (n = 93). The survival of patients with high COX-2 expression (n = 35) was significantly poorer than the survival of patients with low COX-2 expression (n = 58). Log-rank test: *p *= 0.022.

High COX-2 expression was significantly associated with inferior breast cancer-specific survival in ER-negative patients (*p *= 0.001) (Figure 4). We did not find an association between COX-2 and survival in patients with ER-positive tumors in this population (*p *= 0.483). COX-2 was also associated with a significantly decreased survival in patients with high HER2 (HER2-positive) tumors (*p *= 0.022) but not with survival in patients with low HER2 (HER2-negative) breast tumors (*p *= 0.601) (Figure 4). A similar trend was observed when the tumor HER2 status was determined immunohistochemically according to HercepTest™ guidelines. Because FISH results to detect HER2 amplification were not available for this patient cohort, only a HercepTest™ score of 3 was considered positive for tumor HER2 expression in this analysis. There was no significant association of COX-2 with survival in patients being scored negative for HER2 (HercepTest™ score of 0 or 1), but there was a significant poorer outcome among patients with high COX-2 and a positive HercepTest™ score [Log-rank test: *p *= 0.017; HR = 3.2, 95% CI (1.24-9.21) comparing high COX-2 (n = 24) versus low COX-2 (n = 29) in patients with a HercepTest™ score of 3]. Multivariable Cox regression survival analysis at 5-year and 10-year follow-up confirmed these findings (Table 2), indicating that COX-2 was an independent predictor of poor outcome in both ER-negative and HER2-positive breast cancers at 5-year [adj. HR_ER- _= 2.79, 95% CI (1.35-5.78); adj. HR_HER2+ _= 3.39, 95% CI (1.24-9.21)] and 10-year [adj. HR_ER- _= 2.72, 95% CI 1.36-5.41; adj. HR_HER2+ _= 2.84, 95% CI 1.07-7.52] follow-up.

**Table 2:** Effects of high COX-2 expression on breast cancer survival^1^

	**5-Year Multivariable Cox Regression**^3^				**10-Year Multivariable Cox Regression**^3^			
	**H.R**.	95% CI	*p*-value	N	**H.R**.	95% CI	*p*-value	N
All patients	1.82	1.07-3.10	0.028*	184	1.60	0.96-2.65	0.066	184
ER-negative	2.79	1.35-5.78	0.006*	81	2.72	1.36-5.41	0.004*	81
ER-positive	1.26	0.53-2.99	0.598	103	0.96	0.42-2.21	0.920	103
HER2-negative	1.50	0.77-2.94	0.237	121	1.37	0.73-2.56	0.331	121
HER2-positive^2^	3.39	1.24-9.21	0.017*	62	2.84	1.07-7.52	0.036*	62
ER-/HER2-	2.47	1.00-6.10	0.048*	61	2.34	1.01-5.41	0.047*	61
ER-/HER2+	5.95	1.01-34.9	0.048*	20	5.95^3^	1.01-34.9	0.048*	20
ER+/HER2-	0.73	0.21-2.58	0.626	60	0.64	0.18-2.20	0.474	60
ER+/HER2+	2.60	0.47-14.2	0.272	42	1.91	0.42-8.57	0.397	42

^1 ^High COX-2 expression (moderate to strong IHC) versus low COX-2 expression (absent to weak IHC).

^2 ^HER2-positive: moderate to high IHC. ^3 ^Adjusted for age at diagnosis, race, TNM stage, tumor grade, chemotherapy and tumor ER and p53 mutation status. ^3 ^No further deaths after 5 years. *p < 0.05

**Figure 5:**
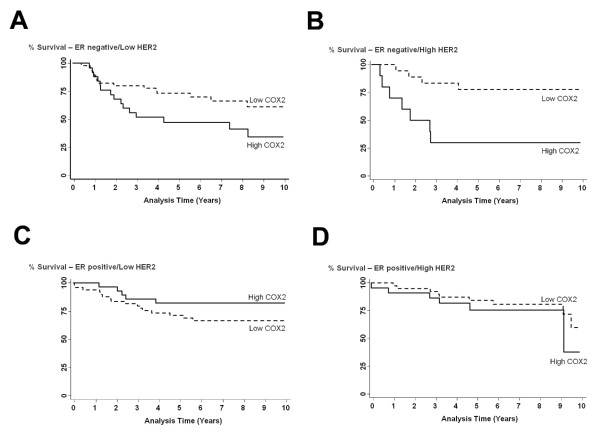
**Effect of ER/HER2 strata on COX-2 associated breast cancer survival**. Kaplan-Meier cumulative breast cancer specific survival curves of (A) ER-negative and low HER2 expressing breast cancer patients by tumor COX-2 status (n = 70). The survival of patients with high COX-2 expression (n = 25) was significantly poorer than the survival of patients with low COX-2 expression (n = 45). Log-rank test: *p *= 0.044. (B) ER-negative and high HER2 expressing breast cancer patients by tumor COX-2 status (n = 28). The survival of patients with high COX-2 expression (n = 10) was significantly poorer than the survival of patients with low COX-2 expression (n = 18). Log-rank test: *p *= 0.006. (C) ER-positive and low HER2 expressing breast cancer patients by tumor COX-2 status (n = 77). The survival of patients with high COX-2 expression (n = 28) was not significantly different from the survival of patients with low COX-2 expression (n = 49). Log-rank test: *p *= 0.182. (D) ER-positive and high HER2 expressing breast cancer patients by tumor COX-2 status (n = 61). The survival of patients with high COX-2 expression (n = 22) was not significantly different from the survival of patients with low COX-2 expression (n = 39). Log-rank test: *p *= 0.383.

While COX-2 remained a predictor of poor outcome in ER-negative patients who were also HER2-negative [adj. HR = 2.34, 95% CI 1.01-5.41], the hazard of poor survival associated with increased COX-2 was highest in ER-negative patients who were HER2-positive [adj. HR = 5.95, 95% CI 1.01-34.9], as shown by univariate (Figure 5) and multivariable analyses (Table 2). ER-/HER2+ tumors belong to a distinct subtype of breast cancer based on its gene expression profile [17]. In ER-positive tumors, no statistically significant association between COX-2 and patient survival was observed, regardless of HER2 status, in our patient population.

### Relationship between COX-2 and the phosphorylation status of Akt, caspase-9 and BAD is dependent on tumor ER and HER2 status

In a previous study of inflammation and breast cancer, we had found a positive correlation between COX-2 expression and increased phosphorylation of Akt, caspase-9 and BAD in breast tumors [9]. Both caspase-9 and BAD are downstream targets of Akt and their phosphorylation inhibits their pro-apoptotic function. Because of our observation that COX-2 is an independent predictor of poor survival in ER-negative and HER2-positive breast tumors, we applied correlation and logistic regression analyses to examine the influence of both the tumor ER and HER2 status on the association between COX-2 and Akt pathway activation in 248 breast tumors.

The correlation analysis revealed that the strongest correlation between increased COX-2 expression and Akt pathway activation is present in tumors that are ER-negative and HER2-positive (pAkt Ser473 *p *= 0.003; pAkt Thr308 *p *= 0.003) (Table 3). In this tumor subtype, COX-2 was also associated with a significantly increased vessel density, as judged by the number of CD31-positive microvessels (*p *= 0.021). The same associations were either weaker or absent (CD31) in all other strata supporting the hypothesis that the poor outcome signature of COX-2 in ER-negative and HER2-positive breast tumors involves Akt pathway activation and increased tumor angiogenesis. Further analysis of the tumor immunohistochemistry by multivariable logistic regression corroborated the results of the Spearman's correlation analysis showing the strongest association between COX-2 expression and Akt pathway activation in ER-negative and HER2-positive breast tumors independent of age at diagnosis, disease stage, race/ethnicity, and neoadjuvant chemotherapy (Table 4). The direction and magnitude of the adjusted odds ratios point to a pathway in which COX-2 induces the phosphorylation of Akt and downstream targets most efficiently in HER2-positive breast tumors, and this effect is increased in an additive manner by an ER-negative tumor status. Furthermore we observed a statistical interaction on survival between COX-2 and pAkt Ser473 (*p *< 0.001), COX-2 and pBad Ser136 (*p *< 0.001), COX-2 and pCasp9 Ser196 (*p *< 0.001), further indicating a significant role of Akt signaling in poor survival of COX-2 expressing tumors.

**Table 3:** Association of COX-2 expression with tumor characteristics by tumor receptor status^1^

	All tumors		ER-negative		ER-positive		Her2 low		Her2 high		ER-negative/HER2 high	
Spearman rank correlation	ρ	*p-value*	ρ	*p*	ρ	*p*	ρ	*p*	ρ	*p*	ρ	*p*
pAkt Ser473	0.23	**<0.001**	0.24	**0.011**	0.22	**0.006**	0.18	**0.024**	0.33	**0.001**	0.50	**0.003**
pAkt Thr308	0.26	**<0.001**	0.25	**0.009**	0.26	**0.001**	0.23	**0.003**	0.36	**<0.001**	0.51	**0.003**
pCasp9 Ser196	0.30	**<0.001**	0.31	**0.001**	0.30	**<0.001**	0.25	**0.001**	0.40	**<0.001**	0.67	**<0.001**
pBAD Ser136	0.20	**0.001**	0.26	**0.008**	0.15	0.063	0.13	0.084	0.31	**0.002**	0.57	**<0.001**
CD31	0.05	0.402	0.10	0.302	0.01	0.85	-0.02	0.799	0.19	0.084	0.41	**0.021**

^1 ^Spearman's correlation coefficient. Calculated with IHC scores 1-4 (negative, weak, moderate, strong) and continuous data (CD31)

**Table 4:** Multivariable logistic regression modeling for association of high COX-2 with increased phosphorylation of Akt, caspase-9, or BAD

	OR*	95% CI	*p*-value	N
**COX-2 and pAkt Ser473**				
All patients	1.52	1.05-2.09	0.024	211
ER_NEG_	1.47	0.84-2.57	0.181	89
ER_POS_	1.57	0.96-2.57	0.072	121
HER2_NEG_	1.33	0.87-2.02	0.183	135
HER2_POS_	2.99	1.09-8.15	0.034	76
ER_NEG_/HER2_POS_	4.87	0.90-26.2	0.065	25
**COX-2 and pAkt Thr308**				
All patients	1.99	1.31-3.02	0.001	207
ER_NEG_	1.85	0.98-3.50	0.057	87
ER_POS_	2.08	1.18-3.63	0.011	119
HER2_NEG_	1.76	1.09-2.86	0.021	134
HER2_POS_	4.25	1.44-12.6	0.009	72
ER_NEG_/HER2_POS_	14.9	1.43-154	0.024	23
**COX-2 and pCasp9 Ser196**				
All patients	1.90	1.36-2.65	0.000	211
ER_NEG_	1.84	1.06-3.22	0.030	89
ER_POS_	2.01	1.31-3.08	0.001	121
HER2_NEG_	1.56	1.06-2.29	0.025	135
HER2_POS_	3.35	1.41-7.91	0.006	76
ER_NEG_/HER2_POS_	16.3	1.72-154.6	0.015	25
**COX-2 and pBAD Ser136**				
All patients	1.61	1.20-2.16	0.001	209
ER_NEG_	1.58	1.04-2.38	0.032	89
ER_POS_	1.66	1.09-2.54	0.019	119
HER2_NEG_	1.42	0.99-2.02	0.051	135
HER2_POS_	2.21	1.22-3.95	0.008	74
ER_NEG_/HER2_POS_	4.10	1.15-14.7	0.030	25

* OR (Odds ratio) adjusted for age at diagnosis, race, TNM stage and receipt of neoadjuvant chemotherapy

## Discussion

In our study of 248 women with incident breast cancer from the Greater Baltimore area, increased expression of COX-2 was associated with decreased breast cancer-specific survival in patients with ER-negative and HER2-positive tumors, respectively. In patients with both ER-negative and HER2-positive tumors, increased tumor COX-2 was associated with the most inferior survival among all patient groups, as judged by the hazard ratio in the multivariable analysis. This was accompanied by increased Akt pathway activation, as judged by the phosphorylation status of Akt and two key downstream targets in the apoptosis pathway. These findings could have implications for COX-2 targeted therapy in breast cancer and suggest that patients with ER-negative and HER2-positive tumors would benefit from a COX-2 targeted therapy with the efficacy of this therapy being strongest in patients with both an ER-negative tumor status and an amplification of HER2 leading to high HER2 expression.

Two recent randomized clinical trials examined the efficacy of the COX-2 inhibitor celecoxib in combination with the aromatase inhibitor, exemestane, in postmenopausal women with hormone sensitive metastatic breast cancer [12,18]. Both trials failed to find a significant clinical benefit with the addition of celecoxib to the exemestane regimen, despite earlier indications in small feasibility studies that there could be increased efficacy for the combination [13,14]. Both trials were performed in predominantly hormone receptor-positive patients. HER2 status information was also available for the Falandry trial [18], with only 4.5% being HER2-positive. The findings in our patient population suggest that the efficacy of COX-2 inhibitors could be quite limited in ER-positive breast cancer, consistent with the trial results by Dirix et al. [12] and Falandry et al [18], suggesting that the benefit of these inhibitors as a therapeutic could be strongest in the ER-negative or HER2-positive disease. Our observation of an association between COX-2 expression with poor survival in ER-negative breast cancer is in agreement with the findings of Witton et al. [19], but are different to the findings of Ristimaki et al. [5], who found that the association of COX-2 expression with distant disease-free survival was restricted to patients with ER-positive or HER2-negative breast cancer. However, there are several differences in the evaluated patients between those in the study of Ristimaki et al and our study, which may contribute to the different findings. All breast cancer patients in our study were recruited as incident cases with surgery, and additionally our study recruited both African-American and European-American patients. The Ristimaki study (recruited 1991-1992) contained solely Finnish patients, with some of them having recurrent disease. There were also differences in receipt of therapy with 47% of patients from our study versus 61% in the Ristimaki study who received postoperative radiotherapy. Of the patients with node negative disease, 75% of our patients received either adjuvant chemotherapy or endocrine therapy, versus 9% in the Ristimaki study. There were also differences in the types of therapies received. In addition, more patients in our study had high grade disease (50%), ER-negative disease (41%) and HER2-positive disease (38%), while in the Finnish study only 30% were diagnosed with high grade breast cancer, 31% had ER-negative disease and 18% had HER2 amplification.

HER2 overexpression/amplification is an established marker of poor prognosis in both early [20] and late [21] stage breast cancer. HER2 overexpression is also associated with an increased risk of metastasis [22,23] and a poor response to chemotherapy in the metastatic setting [24,25]. Several therapies directed at the inhibition of HER2 are currently in use, including the recombinant humanized monoclonal antibodes against HER2, trastuzumbab (Herceptin™, Genentech), and pertuzumab (Omnitarg™, Genentech), and the small molecule tyrosine kinase inhibitor of both HER2 and EGFR Lapatinib (Tykerb™, GlaxoSmithKline) [26]. Akt phosphorylation is associated with HER2 expression in breast cancer [27,28], and it has been shown that HER2 transfection of MCF-7 cells leads to Akt phosphorylation mediated through the PI3K pathway [29]. These observations are consistent with the findings in our study that Akt phosphorylation in breast tumors is significantly associated with HER2 overexpression in the tumors. Additionally Akt phosphorylation has been shown to be associated with COX-2 expression in several studies [2] and COX-2 specific inhibitors were found to disrupt Akt signaling in breast cancer cells [30]. We recently demonstrated that prostaglandin E_2 _(PGE_2_), the most active pro-inflammatory metabolite of COX-2, induces phosphorylation of Akt at Ser473 and GSK-3β at Ser9, a known downstream target of pAkt [9]. Our observation that the correlation of COX-2 with Akt pathway activation is greatest in ER-negative patients, who also have HER2 positivity, are supported by our previous finding that while PGE_2 _phosphorylated Akt at Ser473 and GSK-3β at Ser9 in two breast cancer cell lines, the response was considerably stronger in the ER-negative MDA-MB-231 than the ER-positive MCF-7 breast cancer cells. The result would suggest that ER-negative breast cancer cells may be more sensitive to Akt activation by PGE_2 _than ER-positive breast cancer cells. In summary, existing data suggest that breast cancers, which are ER-negative and overexpress both HER2 and COX-2, may have a more prominent Akt pathway activation, increased resistance to apoptosis, and a higher metastatic potential, which is all consistent with the findings in this study.

Of further interest is that HER2-positive breast tumorigenesis may be modulated in part by COX-2, and *vice versa*. Celecoxib was found to significantly reduce mammary tumor development in HER2/neu-induced experimental mouse models in two separate studies [31,32]. Additionally, tumor multiplicity and size was significantly reduced in the HER2/neu transgenic mice, crossed with a COX-2-deficient background [1]. This suggests that COX-2 significantly contributes to HER2 associated breast tumor development. Wang et al. demonstrated the ability of nuclear HER2 to transactivate COX-2 in colon cancer cells by binding to its promoter region, thus upregulating expression of COX-2 [33], while HER2 has also be shown to up-regulate COX-2 expression through Ras → Raf → MAPK → AP1 mechanisms in breast cancer cells [34]. Both HER2 and COX-2 expression in breast cancer cells lead to the activation of the Akt pathway. Conversely COX-2 and its product PGE_2 _both lead to induction of HER2 gene and protein expression in MCF-7 breast cancer cells [35]. These data indicate that a positive feedback loop exists between COX-2 and HER2 in breast cancer cells.

Our study has strengths and limitations. We conducted the analysis in 248 cases of incident breast cancer, recruiting both European-Americans and African-Americans allowing us to assess the impact of COX-2 on survival in both patient populations. We found that COX-2-related survival was independent of race/ethnicity, indicating that COX-2 inhibition should be equally efficacious in both patient populations. We were also able to assess the implication of the tumor p53 mutational status on the association between COX-2 and breast cancer survival and observed that COX-2-related survival was independent of the p53 mutation status. However, the existing sample size did not allow a more in-depth examination of the effect of race and p53 mutation status on survival in the context of increased COX-2. Furthermore, our immunohistochemical analysis of phosphorylated Akt cannot differentiate between the three Akt isoforms and FISH results to detect HER2 amplification were not available for this patient cohort. We also realize that some clinically important subgroups in our analysis were small, e.g. the number of patients with ER-/HER+ tumors with high COX-2 was n = 10, meaning that results for these subgroups should be interpreted with caution. Thus, additional studies are needed to further corroborate our findings.

## Conclusions

Our study suggests that COX-2 may be a therapeutic target for the treatment of ER-negative breast cancer. A combination of COX-2, HER2, and Akt inhibitors may be particularly efficacious in patients with ER-negative/HER2-positive breast cancer.

## List of Abbreviations

COX-2: cyclooxygenase-2; ER: estrogen receptor α; HR: hazard ratio; CI: confidence interval; PGE_2_: prostaglandin E_2_; TNM stage: tumor-node-metastasis stage; OR: odds ratio; HER2: human epidermal growth factor receptor 2.

## Competing interests

The authors declare that they have no competing interests.

## Author's contributions

SAG conceived and designed the study, performed experiments, participated in data collection, analyzed the data, and drafted the manuscript. RLP contributed to the study design, performed experiments, and analyzed data. LAR participated in study design and carried out experiments. BJB participated in study design, contributed to data collection, and analyzed data. TMD participated in data collection and carried out experiments. DAW participated in study design and manuscript preparation. JEG participated in data analysis and manuscript preparation. HGY performed the pathology and histology evaluation of the tissues and scored the immunohistochemistry. DHL performed the pathology and histology evaluation of the tissues and evaluated immunohistochemistry. SA designed the study, contributed to data collection and analysis, and drafted the manuscript. All authors read and approved the final manuscript.

## Pre-publication history

The pre-publication history for this paper can be accessed here:

http://www.biomedcentral.com/1471-2407/10/626/prepub
